# Routine outcome measurement in adolescents seeking mental health services: standardization of HoNOSCA in Kenyan sample

**DOI:** 10.1186/s12888-021-03438-1

**Published:** 2021-09-06

**Authors:** Grace Nduku Wambua, Manasi Kumar, Fredrik Falkenström, Pim Cuijpers

**Affiliations:** 1 Department of Clinical, Neuro and Developmental Psychology, Amsterdam Public Health Research Institute, Vrije Universiteit Amsterdam, Amsterdam, The Netherlands; 2 Department of Psychiatry, University of Nairobi, Nairobi, Kenya; 3 Department of Behavioral Sciences and Learning, Linköping University, Linköping, Sweden

**Keywords:** Adolescents, Routine outcomes, HoNOSCA, Reliability, Validity

## Abstract

**Background:**

The evaluation of treatment outcomes is important for service providers to assess if there is improvement or not. The Health of the Nation Outcome Scales for Children and Adolescents (HoNOSCA) was developed for this use in child and adolescent mental health services. Outcome measurement in routine mental health services is limited. This paper evaluates the psychometric properties of the self and clinician rated versions of the HoNOSCA for routine use in child and adolescent mental health services in Kenya.

**Methods:**

Using a prospective design, the clinician- and self-rated versions of the HoNOSCA and the Paediatric Symptom Checklist (PSC) were administered at the Youth Centre at the Kenyatta National Hospital in Nairobi. Initial ratings were obtained from adolescents 12-17 years (*n* = 201). A sample of 98 paired ratings with 2 follow-ups were examined for measurement of change over time.

**Results:**

Our findings showed good reliability with the self-rated version of the HoNOSCA score, correlating well with the self-reported version of the PSC (*r* = .74, *p* < .001). Both versions correlated well at follow-up and were sensitive to change. Using factor analysis, the maximum likelihood factoring and Promax rotation resulted in a four-factor structure, which with a Kaiser–Meyer–Olkin measure of sampling adequacy of 0.8 explained 54.74% of total variance.

**Conclusion:**

The HoNOSCA appears to be of value, and easy to use in routine settings. Our findings suggest further investigation with a larger sample.

## Background

There has been an increase in interventions for the management of child and adolescent mental health problems, with reviews showing positive results for psychosocial interventions with children and adolescents [1–5]. One way of assessing the outcome of an intervention is by measuring it during the treatment process. Outcome measures have been identified as essential to demonstrate patient improvement [6] and determine whether these interventions are effective [7]. They also improve clinical practice when it is part of a feedback monitoring system for clinicians by informing clinical decision making and enable the clinician to adjust treatment planning accordingly [8].

The need to evaluate routine health services has led to the development of various outcome measures [8, 9], with the purpose and use of outcome measures depending on the end user [10]. The Health of the Nations Outcome Scales for Children and Adolescents (HoNOSCA) is one of these outcome measures. The HoNOSCA is part of a larger body of work - the Health of the Nation Outcome Scales (HoNOS) developed by Wing et al., [11, 12] in the United Kingdom as a brief mental health measure with the aim of tracking progress towards improving the health and social functioning of mentally ill people. The HoNOS were intended to cover the range of symptoms, behaviours and social difficulties associated with mental illness and can be used in any health setting to measure behaviour, impairment, symptoms, and social functioning in patients [13]. It was noted that the HoNOS scales were not appropriate for use with a younger population, leading to the development of the HoNOSCA by Gowers et al. [14, 15]. The HoNOSCA was first developed as a clinician rated measure to track child and adolescent outcomes in routine care. The self-report version (HoNOSCA - SR) was developed from a criticism that it failed to consider the patient’s perspective [16]. A caregiver version is also available. Although few studies have reported on the self-report version of the HoNOSCA [16, 17], the clinician rated version has been used extensively to regularly monitor the patient’s progress [13]. The HoNOSCA has four subscales relating to: (i) a behaviour (disruptive behaviour, overactivity, self-injury, inattention and substance misuse); (ii) an impairment (physical illness and disability - problems with scholastic or language skills, physical illness or disability problems); (iii) symptoms (psychotic and emotional symptoms - problems associated with hallucinations, delusions or abnormal perceptions, problems with non-organic somatic symptoms or emotional and related symptoms); and (iv) a social ((problems with peer, family relationships, problems with self-care and independence, poor school attendance) [17, 18]. Several studies have examined the psychometric properties of the HoNOSCA [13, 14, 16] and because of its good psychometric properties and ease of use in routine clinical services, it has been translated into different languages [18].

In Kenya, there are few translated and/or culturally validated scales to assess and detect psychopathology especially in children and adolescents. The Child and Behaviour Checklist [19] and Strengths and Difficulties Questionnaire [20] have been commonly used in several studies for the assessment of child and adolescent problems [21–25], but they have not been used to measure outcome during and post treatment. They have also not previously been validated in Kenya.

Evidence on the effectiveness of interventions conducted in mental health services for children and adolescents in Kenya remains limited, in part due to a lack of validated scales. Our study aims to address this issue by evaluating the psychometric properties of the self-report and clinician rated versions of the HoNOSCA for outcome assessment with adolescents seeking mental health services in Kenya.

## Methods

The study was conducted at the Youth Centre, at the Kenyatta National Hospital (KNH), Kenya’s largest teaching and referral hospital. The clinic caters to young people between the ages of 12–24 years. Services offered include mental health, sexual and reproductive health services including Voluntary Testing and Counselling services. The study targeted adolescents between the ages of 12–17 years seeking services for emotional and behavioural problems. Many of these adolescents are referred from academic institutions or by a caregiver. From time-to-time they may self-refer. Adolescents attending the clinics during the months of May 2019 to March 2020 were invited to participate. To be included in the study, patients had to be between 12 and 17 years, both caregiver consent and assent from the adolescents was given. We also invited clinicians working with these adolescents to participate in the study. All participants provided written consent to participate. Patients with moderate to severe intellectual disability, experiencing acute psychotic symptoms and having any unstable medical condition were not included in the study. Our study protocol is described in more detail in Wambua et al. [26].

### Measures

#### Demographic questionnaire

A researcher designed questionnaire that captured relevant demographic variables like age, sex, level of education, reasons for referral, past and present psychiatric history, family (who did they live with, family structure), and social history (substance use, previous trauma history) was used. Psychiatric diagnosis using the Diagnostic and Statistical Manual of Mental Disorders (DSM-IV-TR/V) were retrieved from patient files at the clinic.

#### Health of the nations scale for children and adolescents (clinician-rated HoNOSCA) [14]

The HoNOSCA focuses on clinically significant problems and symptoms the child/adolescent may face, and consists of 15 items, each rated from 0 (no problem) to 4 (for severe problem). It is divided into two sections, where section A relates to the child/adolescent’s condition and covering four subscales: behaviour, impairment, symptoms (psychotic and emotional) and social aspects. Section B relates to the clinician’s assessment of the patients and caregivers’ understanding of the services and management options available to them. The HoNOSCA total score is the sum of the first 13 scales and indicates the severity of the mental health problems. The effectiveness of the HoNOSCA is independent of the type of mental disorder involved, and its psychometric properties has been found to have moderate to good inter-rater reliability, with intraclass correlation coefficients above 0.7 [14, 15] and has validity demonstrated in number of studies [27–30].

#### Health of the nations scale for children and adolescents (self-rated HoNOSCA) [16]

The self-rated HoNOSCA was developed using the 13 items from Section A of the clinician rated HoNOSCA [14]. These items relate to different types of problems the child/adolescent may face within the last 2 weeks, with the adolescent responding, ‘not at all’, ‘insignificantly’, ‘mild but definitely’, ‘moderately’, or ‘severely’. Questions include: *‘Have you been troubled by your disruptive behaviour, physical or verbal aggression’, ‘have you done anything to injure or harm yourself on purpose’, ‘Have you experienced difficulties keeping up with your usual educational attainments and abilities’, ‘Have you found it difficult to look after yourself or take responsibility for your independence’, ‘Have you been troubled by relationships in your family or substitute home’?* Internal consistency was found to be .73 in the initial study by [16], while a Spanish version found alpha of .74 [17].

#### Paediatric symptom checklist - youth version (PSC-youth) [31]

The PSC is often used as a screening measure to assess child and adolescent psychosocial well-being. It is used to identify individuals who may need further evaluation, or as an indicator of psychosocial well-being prior to and following intervention/treatment. This 35-item scale assesses internalizing, externalizing, and attention problems, with the young person rating each item as either never, sometimes, or often. Some of the items include (marking the best that fits the child or adolescent) *complains of aches or pains, are afraid of new situations, act younger than children your age, teases others, blames others for your troubles, less interested in friends, are irritable or angry, absent from school.* A positive score suggests the need for further evaluation. Studies have also indicated strong internal consistency (0.91–0.93) of the scale when inter-item analysis was carried out on the items [32]. In Botswana, Lowenthal et al. [33] found that it had an internal consistency was 0.86 for the PSC-Youth.

### Data collection procedures

The first author trained a research assistant to administer the questionnaires [34]. The research assistant approached potential participants during their first visit at the clinic. The purpose of the study was explained to the adolescents, their caregivers, and clinicians. Those who were interested to participate in the study gave consent (caregivers and clinicians) and assent (adolescents). As English is one of the national languages spoken in Kenya, each participant was then given a set of questionnaires in English. The set of questionnaires consisted of the above-mentioned measures. The clinicians received the original training guide for HoNOSCA in English. The first author was available to help when doubts or questions arose from the participants. Caregivers were not allowed to access the adolescent’s information and clinicians were blinded to the adolescents’ responses.

### Data analysis

Item means, standard deviations, frequencies and percentages were calculated for the socio-demographic variables.

#### Internal consistency

To investigate the reproducibility and consistency of the self and clinician rated versions of the HoNOSCA, reliability coefficients as measured by Cronbach’s alpha were calculated. We used Shrout’s [35] standards for the reliability results: virtually none: 0.00–0.10; slight: 0.11–0.40; fair: 0.41–0.60; moderate: 0.61–0.80; and substantial 0.81–1.0. We also carried out inter-rater reliability between self-report and clinician rated versions using intraclass correlation (ICC, absolute-agreement, 2-way mixed-effects model).

#### Validity

Concurrent validity was examined by comparing the Pearson’s correlation coefficients between totals of the self- and clinician rated HoNOSCA and total PSC scores, as well as the scores on first and second follow-up.

#### Sensitivity to change

Sensitivity to change (test-retest reliability) was assessed in the second/third session (first and second follow-up). A subsample of participants (*N* = 98) was available to be evaluated again with HoNOSCA by both adolescents and clinicians. The ability of the two HoNOSCA versions to reflect changes over time was assessed by observing mean differences between the scores across time points using both Pearson’s correlation coefficient, and *t-*tests.

#### Dimensionality

Before performing factor analysis, the correlation matrix was inspected to check for the strength of correlation and then factorability was tested using exploratory factor analysis using Kaiser-Meyer-Olkin (KMO) measure of sampling adequacy and Bartlett’s test of sphericity. To assess dimensionality underlying the self-rated HoNOSCA items and extract the proper factor structure, we conducted exploratory factor analysis (EFA) based on Maximum Likelihood. Varimax (orthogonal factors with simple structure) and Promax (an oblique rotated factor solution with correlated factors also preserving simple structure) rotation were performed.

## Results

The majority of the participants in our study were female at 54.2% and the mean age of our participants was 15.88 years. The most referrals to the clinic were made by academic institutions (84.2%). Only 11.9% of the participants had previously received prior treatment. Most of the participants (61.2%) experienced psychosocial problems. See Table 1.

**Table 1:** Participant characteristics

		N (%)	Mean (SD)
Sex	Male	92 (45.8)	
	Female	109 (54.2)	
Age			15.88 (1.02)
Academic level	Primary	4 (2.0)	
	Secondary	197 (98.0)	
Referred by	School	170 (84.2)	
	Self	27 (13.4)	
	Family member	1 (0.5)	
	Healthcare worker	3 (1.5)	
Treatment history	None	177 (88.1)	
	Outpatient treatment	24 (11.9)	
Axis I diagnosis*	Depression	3 (1.5)	
	Substance use	46 (22.9)	
	Anxiety	3 (1.5)	
	PTSD	8 (4.0)	
	Suicide ideation	1 (0.5)	
	Conduct disorder	8 (4.0)	
	Conversion disorder	4 (2.0)	
	ADHD	2 (1.0)	
	None	126 (62.7)	
Axis IV*	Interpersonal problems	28 (13.9)	
	Academic problems	30 (14.9)	
	Self-esteem problems	12 (6.0)	
	Psychosocial problems	123 (61.2)	
	Trauma/abuse	8 (4.0)	

* DSM IV-TR/V diagnoses used in the clinics given by clinician and extracted from patient files

The self-rated version of the HoNOSCA and the PSC were completed by all adolescents at intake, and each time they came for follow-up at the clinic. The mean scores at intake were (*N* = 201); M = 10.16 (SD = 7.4) and M = 51.1 (SD = 9.5) respectively. Scores of the PSC suggest that there was significant psychological impairment among all the participants. Clinicians attending to these adolescents also filled in the corresponding HoNOSCA; the mean of these ratings was 7.02 (SD = 5.07).

### Internal consistency

The self-rated HoNOSCA demonstrated acceptable internal reliability with a Cronbach’s alpha of .77. The clinician rated HoNOSCA demonstrated an alpha of .59. The PSC was found to have an alpha of .87. ICC estimates and their 95% confident intervals were calculated based on a mean-rating (k = 3) of the self-report and clinician scores on the HoNOSCA, using absolute-agreement, 2-way mixed-effects model. Good reliability was found between self-report version, with the average ICC measure .827 with agreement definition and 95% confidence interval as .745–.883. The clinician version reported good reliability as well with the average ICC measure .801, (.700–.868). Further, poor reliability was found between the self- and clinician rated versions, with the average ICC measure .305 with agreement definition and 95% confidence interval as −.006–.525.

### Validity of the HoNOSCA

The validity of the self-report HONOSCA was explored by correlating it with the clinician rated version, and PSC. The self-rated HoNOSCA gave a strong correlation of .74 (*p* < .001) with the PSC and weak correlation with the clinician rated HoNOSCA at *r* = .30 (*p* < .001). The clinician rated HoNOSCA gave weak correlations with the PSC at .34 (*p* < .001). The item scores between the two versions were found to be not highly correlated with each other with range of correlations between self-rated (− 0.01–0.49) and clinician rated (− 0.01–0.48) respectively.

### Sensitivity to change

To assess sensitivity to change, we assessed change in scores in the follow up sessions from the intake in a sub-sample (*n* = 98). During intake, the adolescent participants reported higher levels of difficulty compared to the clinician ratings. The correlation of self-report at the first and second follow-up was *r* = .59 (*p* < 0.001) and *r* = .54 (*p* < 0.001) respectively; with an average of 5 days between intake and first follow-up, and 10 between first and second follow-up. The clinician rated version correlated at first follow-up (*r* = .68, *p* < 0.001) and at second follow-up (*r* = .48, p < 0.001) respectively. Independent t-tests were carried out to determine whether the means between sex were different, findings are presented in Table 2. A paired *t*-test was carried out between the initial scores and the first (*t* = 4.297, df = 158, *p* < 0.001). and second follow-up (*t* = 4.705, df = 97, p < 0.001) showing significant differences. Similarly, differences were found in the clinician rated scores at first follow up (*t* = 5.55, df = 157, *p* < 0.001) and second follow-up (*t* = 5.125, df = 97, *p* < 0.001). Thus, the HoNOSCA seems to be sensitive to change.

**Table 2:** Sensitivity to change

			*Self-report*			*Clinician*		
	Self-report	Clinician	Male	Female	t-test	Male	Female	t-test
*First assessment*	10.33 (7.2)	7.38 (4.9)	8.59 (5.2)	11.74 (8.3)	***p = 0.02***	8.84 (4.7)	6.19 (6.2)	***p = 0.01***
*First follow-up*	8.14 (7.7)	6.2 (4.8)	6.61 (5.8)	9.42 (8.8)	*p = 0.06*	7.45 (4.8)	5.15 (4.5)	***p = 0.02***
*Second follow-up*	6.93 (7.6)	4.76 (5.0)	6.57 (7.6)	7.22 (7.7)	*p = 0.67*	5.34 (4.6)	4.28 (5.3)	*p = 0.29*

### Exploratory factor analysis

The suitability of factor analysis was assessed prior to analysis. The Kaiser-Meyer-Olkin KMO = 0.800 and Bartlett’s test of sphericity (476.222, df = 78, *p* < 0.001) justified a dimension reducing procedure such as the factor analysis. The measure of sampling adequacy was > 0.80, so the items could be considered suitable for factor analyses. We carried out a maximum likelihood factor analysis. Our goodness-of-fit test *X*_2_ (df = 32) = 35.544, *p* = .305 suggesting a good fitting model. EFA revealed four components that had eigenvalues greater than one and which explained 54.74% of the variance. As a final EFA model, we retained these four factors. The four factors were related to relationship problems (1,3,10,11,12,13), severe psychiatric symptoms (3,7,9), school problems (2,5,11) and physical problems (6). Table 3 reproduces the factor pattern of EFA structure with Promax-rotated factors.

**Table 3:** Exploratory factor analysis (Promax-rotated factors)

	Factor			
	1	2	3	4
1. Disruptive, antisocial or aggressive behaviour	0.409			
2. Over-activity attention and concentration			0.473	
3. Non accidental self-injury	0.321	0.521		
4. Alcohol, substance/solvent misuse				
5. Scholastic or language skills			0.836	
6. Physical illness or disability problems				0.989
7. Hallucinations and delusions		0.703		
8. Non-organic somatic symptoms				
9. Emotional and related symptoms		0.659		
10. Peer relationships	0.732			
11. Self-care and independence	0.306		0.341	
12. Family life and relationships	0.591			
13. Poor school attendance	0.362			

## Discussion

While the HoNOSCA is widely implemented and continues to garner significant recognition, these studies have all been carried out in the developed world. It has not been used or validated in any LMIC context. Our study found both versions of the HoNOSCA easy to use and reliable to assess global severity of mental health problems experienced by children and adolescents seeking services in the youth clinic at KNH.

Internal consistency of the self-rated HoNOSCA was at .77, which suggests that the different items carried independent weight, similar to Ballesteros-Urpi et al. [17] who found alpha of .74 and .76 on the self-rated version. Our findings on the clinician version showed poor internal consistency (.58), which was similar to other studies that found low alpha at .45 [36] and .65 [37]. Inter-rater reliability for both scales was found to be strong and similar to those found in other studies, which range from 0.72 to 0.96 [17, 18, 27–29].

Concurrent validity between the self-rated version and the PSC were moderate. The poor correlations between clinician rated and self-reported HoNOSCA or PSC scores were similar as in other studies. The initial study validating the self-report version [16] found it to be weak when compared with the SDQ. Other studies also found poor correlations between the clinician rated HoNOSCA and other adolescent self-reports [17, 22, 29]. The different item scores poorly correlated with each other on both versions and this was found to be similar with the original study by Gowers et al. [14] whose correlations range of correlations were 0.01–0.41, suggesting that the HoNOSCA is unidimensional scale. These poor coefficients suggest that there is limited comparability between the two versions of the scale warranting further research with specific tools for adolescents and clinicians. The low concurrent validity of clinician rated HoNOSCA, requires that we carry out further validation studies with scales geared for clinicians.

Change in the scores at follow-up were moderately correlated with initial scores on both scales of the HoNOSCA. Similar with other studies, our findings show that both versions are sensitive to change, indicating that they can be particularly helpful for use in child and adolescent mental health services [14, 16, 17, 27].

We found a four-factor solution, our factor analysis did not confirm the areas of functioning as hypothesised a priori by Gowers et al. [14] or as evidenced by Tiffin and Rolling [38]. Further investigation is required to assess the factor structure in our local context as sub-scales may not translate easily across cultures.

### Strengths and limitations

To our knowledge, this is the first study to assess the psychometric properties of the HoNOSCA in a LMIC context, specifically Africa. A strength of our study is that it looked at both clinician and self-rated versions concurrently. Some limitations of our study included: the test-retest took place at different times with a mean of 5 days (S.D = 4.72) between initial assessment and first follow-up and 10 days (S.D = 15.38) between first and second follow-up. This may have influenced internal consistency of the different versions. Our clinicians (*n* = 69) varied in academic background and level of expertise with some having diploma in counselling, bachelor’s in counselling/nursing, master’s in counselling/ clinical psychology/ psychiatric social-work/ nursing and doctorate level, together with the large number of raters may have influenced the internal consistency of the clinician rated HoNOSCA. Another limitation is that our study only recruited patients seeking outpatient services, and with less severe or complex mental health problems. The study was also carried out in an urban area and being a public hospital may draw patients with high levels of psychosocial disadvantage. The length of treatment was also not suitable enough to assess outcomes.

## Conclusion

The HoNOSCA has proven to be easy to use in routine care and useful to achieve outcome measurements in child and adolescent mental health services. We would benefit from further testing of HoNOSCA in our context with a larger sample, and strict parameters between sessions. Internal consistency and concurrent validity of the clinician version was low warranting further evaluation.

## Data Availability

Data is available upon request from the authors.

## Abbreviations

CAMH: Child and adolescent mental health
DSM IV/V: Diagnostic and Statistical Manual of Mental Disorders
PSC: Paediatrics Symptom Checklist - Youth version
HoNOS: Health of the Nation Outcome Scales
HoNOSCA: Health of the Nation Outcome Scales for Children and Adolescents
KNH: Kenyatta National Hospital
ICC: Intraclass coefficient
KMO: Kaiser-Meyer-Olkin
EFA: Exploratory Factor Analysis
